# Laccase Activity in CTAB-Based Water-in-Oil Microemulsions

**Published:** 2016

**Authors:** Maryam Azimi, Nastaran Nafissi-Varcheh, Mohammad Ali Faramarzi, Reza Aboofazeli

**Affiliations:** a *Department of Pharmaceutical Biotechnology, School of Pharmacy, Shahid Beheshti University of Medical Sciences, Tehran, 19919-53381, Iran. *; b *Department of Pharmaceutical Biotechnology, Faculty of Pharmacy & Biotechnology Research Center, Tehran University of Medical Sciences, Tehran 14176-14411, Iran.*; c *Department of Pharmaceutics, School of Pharmacy & Protein Technology Research Center, Shahid Beheshti University of Medical Sciences, Tehran, 19919-53381, Iran.*

**Keywords:** Laccase, Enzyme activity, W/o microemulsions, CTAB

## Abstract

The aim of this study was to develop a microemulsion system as a medium for laccase-catalyzed reactions. Phase behavior studies were conducted by constructing partial pseudo-ternary phase diagrams for systems comprising of cetyltrimethylammonium bromide (CTAB), various organic solvents as the oil phase (i.e., hexane, cyclohexane, heptane, octane, isooctane, toluene, isopropyl myristate), two co-surfactants (i.e., 1-butanol and 1-hexanol) and citrate buffer solution, at various surfactant/co-surfactant weight ratios (*R*_sm_). A monophasic, transparent, non-birefringent area (designated as microemulsion domain) was seen to occur in some phase diagrams along the surfactant/organic solvent axis, the extent of which was dependent mainly upon the nature of co-surfactant and *R*_sm_. On each phase diagram, three different water-in-oil (w/o) microemulsion systems with less than 50 wt% surfactant mixture and less than 20 wt% of aqueous phase were selected for laccase loading and activity measurements. Results revealed that the catalytic activity of laccase in CTAB-based w/o microemulsions decreased considerably, compared with its activity in the buffer solution, the extent of which depended upon the type of component and their compositions in the microemulsions. It was suggested that the conformational changes due to the electrostatic interactions between the cationic head group of CTAB and the negative enzyme might be the reason for the reduction of laccase activity, once entrapped in the microemulsion.

## Introduction

Laccases (benzenediol: oxygen oxidoreductase, EC 1.10.3.2) belong to a family of copper-containing oxidases which are widespread in fungi and have been isolated from higher plants, insects and some bacteria. Laccases catalyze the reduction of molecular oxygen to water and simultaneously conduct single-electron oxidation of various inorganic and aromatic substrates, particularly phenols (1-4). These enzymes have attracted more attentions since they are generally eco-friendly, they only require oxygen as the co-substrate, they release water as the only by-product and they a wide substrate range. Laccases have been investigated extensively and found wide application potential in various areas, including environmental pollution control, textile industry, biosensors, food industry, pharmaceutical industry and organic synthesis (5, 6).

Water-in-oil (w/o) microemulsions are considered as an interesting alternative to conventional aqueous media for conducting enzymatic reactions (7). W/o microemulsions are self-organized aggregates of surfactant molecules (sometimes with co-surfactants) which are dispersed in a non-polar organic phase and contain nanometer-sized water cores. These systems are homogeneous, thermodynamically stable, isotropic and optically transparent. The rationale of using these systems as a media for enzymatic reactions is the potential advantages and possibilities they provide. W/o microemulsions can host biomolecules such as enzymes through homogenous solubilization of the biomolecule in their water core and protect them from unfavorable contact with the surrounding organic phase. They also provide an enormous water-oil interfacial area through which catalytic reactions with water-insoluble substrates can occur. Microemulsions are formed spontaneously and therefore the effect of shear stress forces on the sensitive enzyme structure, due to the mixing of the reaction system, is reduced. In addition to these advantages, microemulsion droplets can entrap the enzyme at a molecular scale and the dynamic and flexible characters of the droplets are profitable to the enzyme reactivity. It has also been reported that once these nanostructures are used as the reaction media, the activity and stability of the enzymes may be significantly improved and can be controlled mainly by the concentration of water in these media (8-13). W/o microemulsions also provide an interesting alternative to normal organic solvents in enzyme catalysis with hydrophobic substrates. Catalytic reactions with water insoluble substrates can occur at the large internal water-oil interface (14).

Several enzymatic reactions with different enzymes have been conducted and studied in microemulsions. Examples are hydrolases (lipases, esterases, glucosidases, and proteases), oxidoreductases (peroxidases, oxygenases, and dehydrogenases), and transferases (kinases) (15). The catalytic activity of enzymes in microemulsion systems is highly dependent upon the composition and structure of the microemulsion. Therefore, in an attempt to determine the suitable reaction medium, it is important to evaluate the enzyme activity as a function of microemulsion composition (14).

Cetyltrimethylammonium bromide (CTAB)-based w/o microemulsion systems have been investigated as media for enzyme-catalyzed reactions. Rees and Robinson used *Chromobacterium viscosum* (CV) lipase, solubilized in water-in-oil microemulsions based on CTAB, for an ester synthesis. They demonstrated that the stability of CV lipase in all the CTAB microemulsions studied was significantly higher than that observed in aqueous buffer at the same pH and temperature (16). Giuliani and his co-workers have reported the synthesis of pentyl ester of ferulic acid catalyzed by *Aspergillus niger* feruloyl esterases (FAEA) using CTAB/hexane/pentanol water-in-oil microemulsions. Their results depicted that the enzyme stability was significantly higher in the microemulsion than that in an aqueous solution, suggesting that the microemulsion could keep FAEA in the more active conformation, due to the conformational constraints imposed by the micellar system on the protein structure (17). The enzymatic activity of Lipase VII from *Candida rugosa* was also studied in the quaternary water-in-oil microemulsion CTAB/water/pentanol/hexane. The data obtained has confirmed a marked influence of the extent of interfacial surface on the catalytic efficiency of lipase (18). An improved activity of Horseradish peroxidase (HRP) in the w/o microemulsion of CTAB with an increase in *n*-hexanol concentration and water/surfactant weight ratio has been observed, which was attributed to the increased interfacial area of the microemulsions (19). There are some reports considering the positive effect of the CTAB on the laccase activity. It has been shown that CTAB could significantly improve the activity and stability of the laccase from the white-rot fungus *Meripilus giganteus *(20) and the bacterial laccase purified from *Bacillus tequilensis *SN4 (21).

The aim of this study was to develop a CTAB-based microemulsion medium as a microreactor for laccase. It was hypothesized that the presence of this surfactant can enhance the activity of laccase from *Trametes versicolor.* As the first step, phase behavior of systems composed of various organic solvents (as oil), two short-chain alcohols (as co-surfactants) and buffer solution, at various surfactant/co-surfactant weight ratios (*R*_s__m_) were studied through the construction of phase diagrams. In the second step, the activity of laccase entrapped in these systems were investigated, using ABTS as the substrate.

**Table 1:** Composition of laccase-loaded, CTAB/1-butanol microemulsion systems at the *R*_sm_ of 1:1 (selected from phase diagrams) and the corresponding results of the enzyme activity measurements (n = 3; SD < 0.04

**Activity ** **(μmole.min** ^-1^ **.mg** ^-1^ **) **	**Surfactant (wt%)**	**Oil (wt%) **	**Water (wt%)**	**point on phase diagram**	**Oil type**	** system**
0.469	25.89	60.41	13.70	A	Hexane	S1
0.434	34.52	51.78	13.70	B	Hexane	S2
0.626	40.32	40.33	19.35	C	Hexane	S3
0.333	26.26	61.27	12.47	A	Cyclohexane	S4
0.319	35.01	52.52	12.47	B	Cyclohexane	S5
0.331	41.72	41.72	16.56	C	Cyclohexane	S6
0.354	26.04	60.75	13.21	A	Heptane	S7
0.521	34.72	52.07	13.21	B	Heptane	S8
0.792	40.81	40.81	18.38	C	Heptane	S9
0.301	26.13	60.97	12.90	A	Octane	S10
0.557	34.84	52.26	12.90	B	Octane	S11
1.011	40.91	40.91	18.18	C	Octane	S12
0.342	26.22	61.17	12.61	A	*iso*-octane	S13
0.504	34.90	52.35	12.75	B	*iso*-octane	S14
0.677	41.29	41.29	17.42	C	*iso*-octane	S15

**Table 2:** Composition of laccase-loaded, CTAB/1-butanol microemulsion systems at the *R*_sm_ of 1:2 (selected from phase diagrams) and the corresponding results of the enzyme activity measurements (n = 3; SD < 0.05

**Activity** **(μmole.min** ^-1^ **.mg** ^-1^ **)**	**Surfactant (wt%)**	**Oil (wt%) **	**Water (wt%)**	**point on phase diagram**	**Oil type**	** system**
0.663	27.98	65.29	6.73	A	Hexane	S16
0.607	36.24	54.35	9.41	B	Hexane	S17
0.262	42.54	42.54	14.92	C	Hexane	S18
0.368	28.15	65.68	6.17	A	Cyclohexane	S19
0.354	36.49	54.73	8.78	B	Cyclohexane	S20
0.132	43.23	43.23	13.54	C	Cyclohexane	S21
0.764	27.98	65.29	6.73	A	Heptane	S22
0.506	36.24	54.35	9.41	B	Heptane	S23
0.262	42.54	42.54	14.92	C	Heptane	S24
0.811	28.03	65.41	6.56	A	Octane	S25
0.670	36.19	54.29	9.52	B	Octane	S26
0.296	42.70	42.71	14.59	C	Octane	S27
0.950	28.06	65.47	6.47	A	*iso*-octane	S28
0.597	36.14	54.22	9.64	B	*iso*-octane	S29
0.272	42.78	42.78	14.42	C	*iso*-octane	S30

**Table 3:** Composition of laccase-loaded, CTAB/1-hexanol microemulsion systems at the *R*_sm_ of 1:1 (selected from phase diagrams) and the corresponding results of the enzyme activity measurements (n = 3; SD < 0.05).

**Activity** **(μmole.min-1.mg-1)**	**Surfactant (wt%)**	**Oil (wt%)**	**Water (wt%)**	**point on phase diagram**	**Oil type**	**system**
0.556	25.89	60.41	13.70	A	Hexane	S31
0.575	33.99	52.94	13.07	B	Hexane	S32
0.350	41.01	41.01	17.98	C	Hexane	S33
0.231	26.42	61.65	11.93	A	Cyclohexane	S34
0.344	35.28	52.91	11.81	B	Cyclohexane	S35
0.316	41.64	41.64	16.72	C	Cyclohexane	S36
0.216	26.04	60.75	13.21	A	Heptane	S37
0.542	34.78	52.17	13.05	B	Heptane	S38
0.529	40.91	40.91	18.18	C	Heptane	S39
0.103	26.08	60.86	13.04	A	Octane	S40
0.631	34.90	52.35	12.75	B	Octane	S41
0.629	41.29	41.29	17.42	C	Octane	S42
0.463	26.04	60.75	13.21	A	*iso*-octane	S43
0.583	35.07	52.60	12.33	B	*iso*-octane	S44
0.523	41.29	41.29	17.42	C	*iso*-octane	S45

**Table 4:** Composition of laccase-loaded, CTAB/1-hexanol microemulsion systems at the *R*_sm_ of 1:2 (selected from phase diagrams) and the corresponding results of the enzyme activity measurements (n = 3; SD < 0.03

**Activity** **(μmole.min-1.mg-1)**	**Surfactant (wt%)**	**Oil (wt%)**	**Water (wt%)**	**point on phase diagram**	**Oil type**	**system**
0.317	27.96	65.23	6.81	A	Hexane	S46
0.213	37.38	56.07	6.55	B	Hexane	S47
0.083	45.29	45.29	9.42	C	Hexane	S48
0.277	28.74	67.06	4.20	A	Cyclohexane	S49
0.163	37.59	56.38	6.03	B	Cyclohexane	S50
0.052	45.70	45.71	8.59	C	Cyclohexane	S51
0.475	28.01	65.31	6.68	A	Heptane	S52
0.259	37.41	56.11	6.48	B	Heptane	S53
0.092	45.40	45.41	9.19	C	Heptane	S54
0.424	27.98	65.29	6.73	A	Octane	S55
0.233	37.41	56.11	6.48	B	Octane	S56
0.103	45.35	45.35	9.30	C	Octane	S57
0.421	28.01	65.35	6.64	A	*iso*-octane	S58
0.269	37.41	56.11	6.48	B	*iso*-octane	S59
0.087	45.40	45.41	9.19	C	*iso*-octane	S60

**Figure 1:**
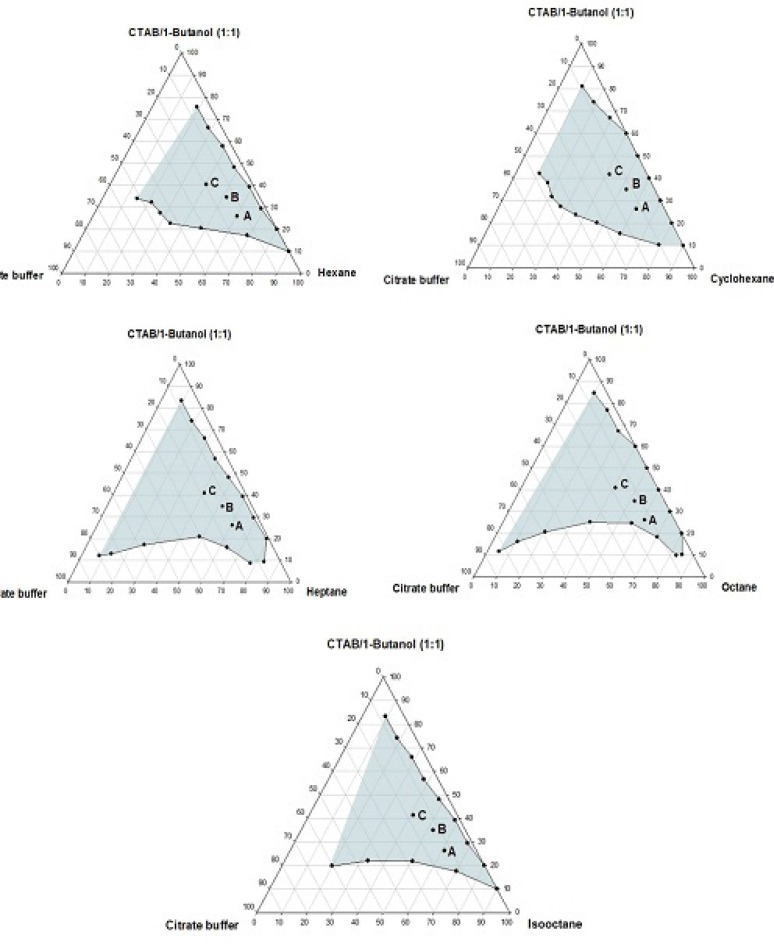
Phase diagrams of the quaternary systems containing CTAB/1-butanol/organic solvents/aqueous citrate buffer solution, constructed at the *R*_sm_ of 1:1. The shadow area represents the transparent, non-birefringent, isotropic area, designated as microemulsion domain

**Figure 2:**
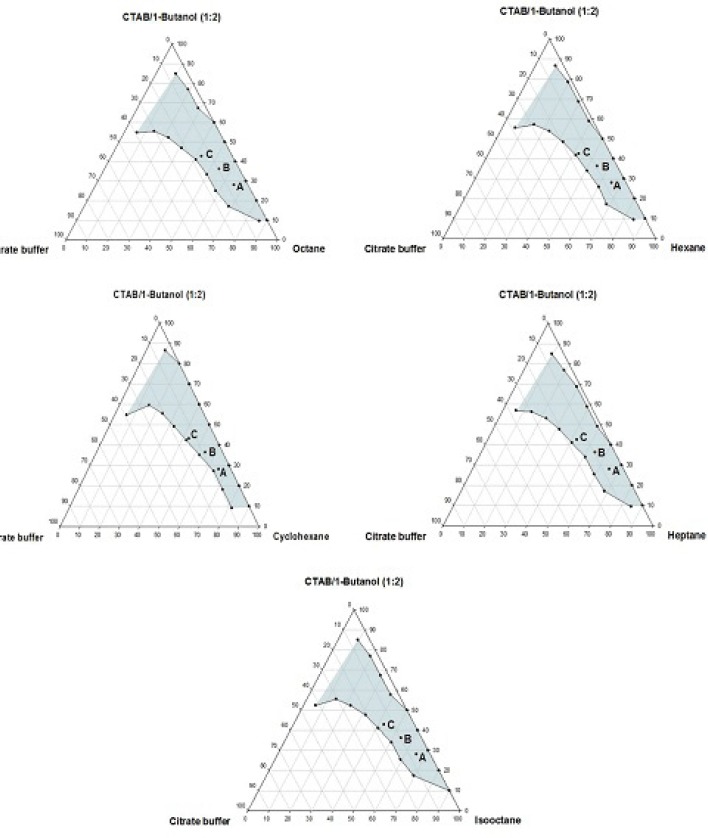
Phase diagrams of the quaternary systems containing CTAB/1-butanol/organic solvents/aqueous citrate buffer solution, constructed at the *R*_sm_ of 1:2. The shadow area represents the transparent, non-birefringent, isotropic area, designated as microemulsion domain

**Figure 3:**
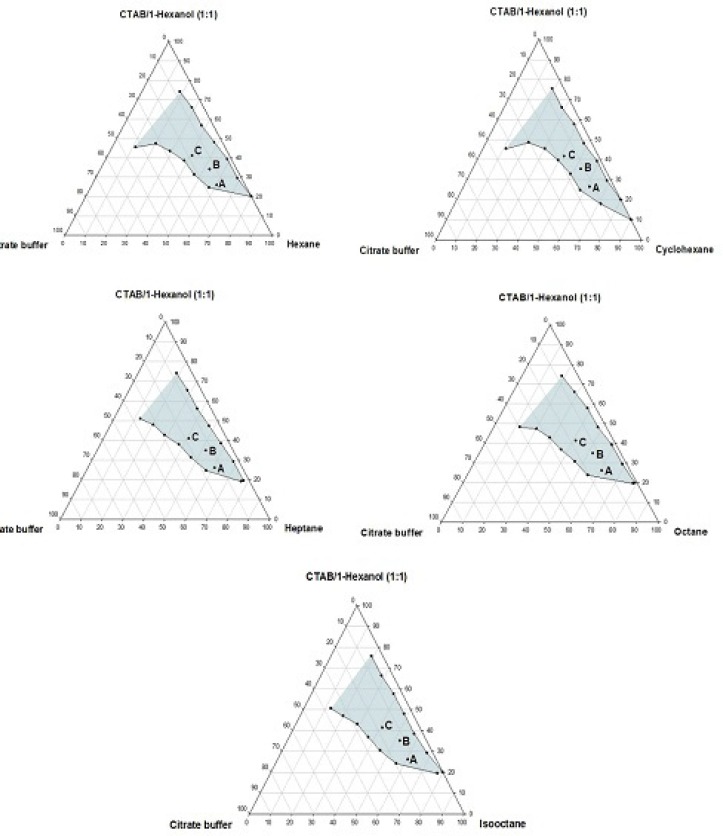
Phase diagrams of the quaternary systems containing CTAB/1-hexanol/organic solvents/aqueous citrate buffer solution, constructed at the *R*_sm_ of 1:1. The shadow area represents the transparent, non-birefringent, isotropic area, designated as microemulsion domain

**Figure 4:**
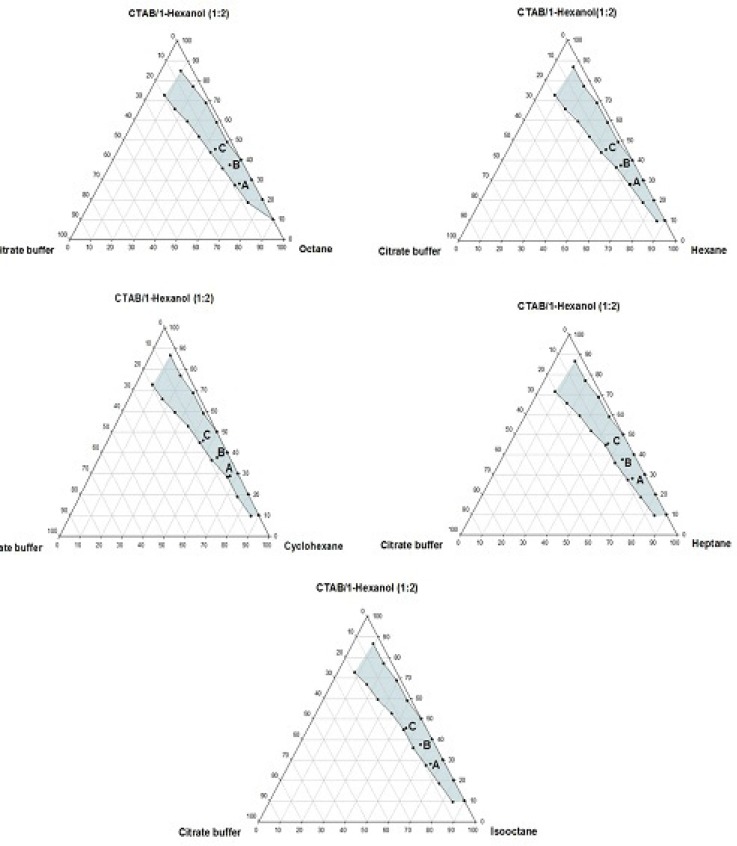
Phase diagrams of the quaternary systems containing CTAB/1-hexanol/organic solvents/aqueous citrate buffer solution, constructed at the *R*_sm_ of 1:2. The shadow area represents the transparent, non-birefringent, isotropic area, designated as microemulsion domain

## Experimental

*Materials*

Laccase from *Trametes versicolor*, cetyltrimethylammonium bromide (CTAB) and octane were purchased from Sigma-Aldrich (St Louis, MO, USA). The substrate 2,2′-Azino-bis(3-ethylbenzothiazoline-6-sulfonic acid) diammonium salt (ABTS) was obtained from Sigma-Aldrich (Steinheim, Germany). 1-Butanol, 1-hexanol, hexane, heptane, *iso*-octane, toluene and *iso*-propyl myristate were provided from Merck Chemical Company (Darmstadt, Germany). Cyclohexane was obtained from Titran Chemical Company (Iran).

*Construction of pseudo-ternary phase diagrams*

Appropriate amounts of various organic solvents (including hexane, cyclohexane, heptane, octane, *iso*-octane, toluene, *iso*-propyl myristate), CTAB (as the cationic surfactant) and two co-surfactants (including 1-butanol and 1-hexanol) were weighed into screw-capped vials and the samples were stirred at room temperature until a clear solution was obtained. Pseudo-ternary phase diagrams were constructed by titrating these samples with aliquots of 0.1 M citrate buffer (pH = 5) at room temperature and stirring for a sufficiently long time to attain the equilibrium. The endpoint of the titration was determined when the system became turbid. The course of each titration was also monitored both visually for clarity and through cross polaroids in order to determine the boundaries of any birefringent liquid crystalline phase. The phase behavior of the mixtures investigated was mapped on triangle phase diagrams with the top apex representing a specific surfactant-co-surfactant weight ratio (*R*_sm _of 1:1, 1:2 and 2:1) and the other two apices representing the organic solvent (oil) and aqueous buffer phase. In this study, all mixtures produced optically transparent, homogenous, non-birefringent isotropic solutions were termed microemulsions and the corresponding areas on the phase diagrams were designated as microemulsion domains.

*Preparation of laccase-loaded w/o microemulsions*

Three microemulsion points (w/o) positioned at the buffer phase-poor part of the phase diagrams, within the range of 20-50 wt% of the surfactant mixture and 10-20 wt% of the aqueous buffer phase were selected in an attempt to load the enzyme (laccase) and measure its activity. Laccase-loaded microemulsions were prepared by the spontaneous emulsification technique. Firstly, appropriate amounts of the oil, surfactant and co-surfactant were mixed. To 1.8 mL of the resultant mixture in a test tube, 50 µL of the enzyme solution (0.5 mg/mL in citrate buffer, 0.1 M, pH = 5) was then injected. If needed, an additional amount of the buffer solution was added to attain the desired water concentration level. The whole mixture was gently shaken for a few seconds in order to obtain a macroscopically homogenous solution.

*Measurement of enzyme activity*

The catalytic activity of laccase was investigated with ABTS as the substrate .ABTS is oxidized by laccase to its corresponding cation radical (ABTS^●+^) which shows spectrophotometric absorbance at 420 nm and has an extinction coefficient (ε) of 36000 M^-1^cm^-1^. To initiate the enzymatic reaction, 0.15 mL of the substrate ABTS (from 15 mM stock solution in citrate buffer, 0.1 M, pH = 5.0) was added to laccase-loaded microemulsions as described before. The reaction rates were determined by spectrophotometrically monitoring the absorbance change over time at 420 nm, due to the appearance of ABTS^●+^ in the first 10 minutes, using UV–visible spectrophotometer (ScanDrop® spectrophotometer, Analytik Jena, Germany). The laccase activity was calculated using the molar extinction coefficient of ABTS (ε_420_ = 36000 M^−1^ cm^−1^) (22). The activity was defined as the amount of ABTS oxidized per minute by 1 mg of the enzyme at pH 5.0 and 40 °C. The activity measurement was performed in triplicate.

*Statistical analysis*

Data are reported as mean ± SD. Statistical analysis of differences between the samples was performed using one-way ANOVA and an appropriate post-test, if necessary. A 0.05 level of probability was taken as the level of significance.

## Results and Discussion

Microemulsions are mixtures of three components, namely water, a hydrocarbon and a single surfactant. Anionic surfactants, such as sodium (bis-2-ethyl-hexyl) sulfosuccinate which is an anionic surfactant widely known as Aerosol-OT (or AOT), are able to solubilize large quantities of water in the organic phase and establish stable water-in-oil microemulsions without the aid of a co-surfactant. (23, 24). This means that co-surfactants are not a prerequisite for the formation of microemulsions (25). Most cationic surfactants (quaternary ammonium salts), however, have no appropriate HLB to form reversed micelles in alkanes at room temperature and therefore, a co-surfactant is required (26). On the other hand, reversed micelles formed by cationic surfactants are smaller than those formed by anionic surfactants, thus, a co-surfactant is added to the system to enlarge the micelle size when a cationic surfactant is used (25, 27-29). It has been suggested that co-surfactant molecules are inserted between the molecules of the surfactant (30), reduce the strong repulsive interaction between the surfactant head groups and arrange the big surfactant molecules in a loose manner, thus allowing their close packing and formation of the reversed micelle aqueous core (27, 31), followed by dissolution of surfactant in solvent (27, 32). 

Phase behavior studies were conducted by constructing partial pseudo-ternary phase diagrams for systems comprising of cetyltrimethylammonium bromide (CTAB), various organic solvents as the oil phase, two co-surfactants and citrate buffer solution, at various surfactant/co-surfactant weight ratios (*R*_sm_) (Figures 1-4). In general, the following generalizations can be made about the systems examined:

a) Results showed that CTAB was not capable of stabilizing w/o microemulsions in the absence of the co-surfactants used in this investigation.

b) It was also observed that CTAB was not capable of producing isotropic solutions with *iso*-propyl myristate and toluene, even in the presence of a short-chain alcohol.

c) Regardless of the type of co-surfactant, microemulsions were found to form using hexane, cyclohexane, heptane, octane and *iso*-octane as the organic phase, at the *R*_sm_ of 1:1 and 1:2. No microemulsion region was observed on the phase diagrams, at the *R*_sm_ of 2:1.

d) The extent of the isotropic area was found to be considerably dependent upon both the nature of co-surfactant and *R*_sm_.

e) Irrespective of the type of organic solvent and *R*_sm_, the extent of the microemulsion region was significantly larger in the presence of 1-butanol, compared to the systems composed of 1-hexanol.

f) Among the systems studied, the largest microemulsion domain was observed in CTAB/1-butanol (*R*_sm_ of 1:1) mixture.

g) Regardless of the nature of co-surfactant and organic solvent, the extent of microemulsion area at *R*_sm_ of 1;1 was greater than that obtained at *R*_sm_ 1:2.

Laccase activity in 60 different microemulsion systems was represented by the appearance of ABTS^●+^ which showed a strong spectrophotometric absorbance at 420 nm. All microemulsions systems were prepared by using 20-50 wt% of surfactant/co-surfactant mixture and 10-20 wt% of an aqueous buffer phase. The composition of microemulsions studied and the results of the laccase activity measurements are tabulated in Tables 1-4.

Reverse micelles or w/o microemulsions have found wide applications in enzymology and biocatalysis. Microemulsions were introduced to solubilize enzymes in organic solvents in order to shelter and protect the enzyme from solvent effects and provide a unique microenvironment for the enzymes to react with water insoluble or poorly soluble substrates present in the organic phase (33). There are few reports in the literature, describing the use of w/o microemulsion systems for conducting laccase reactions. The oxidation of hydrophobic organic pollutants, bisphenol A and chlorophenols, by laccase entrapped in an AOT-based reversed micellar system has been investigated by Okazaki and his co-workers (34). They showed that the laccase/reverse micellar system could effectively catalyze the oxidation reaction in isooctane as the organic solvent, while the lyophilized laccase exhibited no catalytic activity in nonaqueous media. They also evaluated the influence of various parameters in an attempt to optimize the reaction conditions, including pH in the water pools of reverse micelles, the concentration of laccase, and the degree of surfactant hydration on the laccase activity in organic media. Results demonstrated a strong pH-dependency in organic media with the optimum activity at pH = 5. In another research, a laccase complexed with surfactants has been used for oxidative degradation of phenolic environmental pollutants in organic media (35). It was concluded that by appropriately adjusting the water content of the reaction medium (water core of reverse micelles), the catalytic activity of the surfactant (AOT) – laccase complex in isooctane could be significantly enhanced. This study revealed that the surfactant–laccase complex had little activity towards the oxidative reaction of bisphenol A in water-saturated isooctane (i.e. 0.0055% [v/v] water), whereas effectively catalyzed the same reaction in isooctane containing 4% (v/v) water (over the maximum water solubility). By addition of a redox mediator in the reaction medium using reverse micelles, the surfactant–laccase complex investigated in this study was also capable of catalyzing chlorophenols, once a redox mediator was simultaneously added into the reaction medium using reverse micelles.

Although most of the investigations on enzyme catalytic reactions in reverse micelles or microemulsions have been carried out by using anionic or non-ionic surfactants, however, there are some reports in the literature regarding the application of cationic surfactants for the preparation of w/o microemulsions, in an attempt to evaluate the activity of α-chymotrypsin, trypsin, lipase and hydrogenase (15). As mentioned earlier, the aim of this study was to develop a CTAB-based microemulsion medium as a microreactor for laccase from *Trametes versicolor, *using ABTS as the substrate. Five different organic oils and two short-chain alcohols (as co-surfactants) were selected for the partial phase behavior studies. On each phase diagram constructed, three different points in the w/o microemulsion domain were selected for laccase activity assessment. As depicted in Figures 1-4. by moving from point A to point C, the weight percent of buffer phase and surfactant mixture were increased, while the content of the organic solvent simultaneously was decreased. In general, the following generalizations can be made, regarding the laccase activity results in CTAB-based microemulsion systems studied:

a) At the *R*_sm_ of 1:2, in the presence of 1- butanol, the enzyme activity declined from point A to C in all systems investigated. The same trend was observed at the *R*_sm_ of 1:1, except in systems prepared with cyclohexane.

b) At the *R*_sm_ of 1:2, in the presence of 1- hexanol, the enzyme activity decreased from point A to C in all systems studied. However, when the co-surfactant concentration was decreased (*R*_sm_ of 1:1), the activity first exhibited an increase from point A to B and then showed a decrease from point B to C.

c) Regardless of the type of organic solvent, at the *R*_sm_ of 1:2, the activity was found to rise in the presence of hexanol, compared to the systems composed of 1-butanol. 

d) At the *R*_sm_ of 1:1, the highest activity was seen at points C and B in the presence of 1-butanol and 1-hexanol, respectively. 

e) In the presence of 1-butanol as the co-surfactant, among the systems studied, the least and the highest laccase activity were observed in CTAB/cyclohexane and CTAB/octane systems, respectively.

f) In the presence of 1-hexanol as the co-surfactant, among the systems studied, the least and the highest laccase activity were observed in CTAB/cyclohexane and CTAB/*iso*-octane systems, respectively.

Our results revealed that laccase has lost some of its activity once incorporated in CTAB-based w/o microemulsions, compared to its activity in the buffer medium (15.71 μmole.min^-1^.mg^-1^). This effect may be attributed to the conformational changes in the laccase. Investigations on enzyme activity in aqueous surfactant solutions have demonstrated that surfactants could influence the catalytic properties of enzymes. Electrostatic and hydrophobic interactions between an enzyme and a surfactant has been reported in the literature. The former takes place between the head group of the surfactant molecule with a charged amino acid, while the latter involves the interaction between the alkyl chain of the surfactant and the hydrophobic residues of the enzyme. It has been proposed that these interactions can induce a change in the enzyme structural conformation (36). Yang and his co-workers in their investigation on the effect of non-ionic and ionic surfactants on the stability and activity of tyrosinase isolated from *Agaricus bisporus* have compared the kinetic parameters and demonstrated that AOT could increase the catalytic activity of the enzyme, whereas CTAB could cause its deactivation (37).

In our previous study on the laccase activity in the presence of various surfactants, we showed that CTAB caused severe inactivation of the enzyme. We proposed that this effect may be probably due to the interaction between CTAB and laccase and, in turn, to the modification of the enzyme structure (38). The isoelectric point of laccase isolated from *T. versicolor* is about 3.9 (39). Therefore, laccase carries a net negative charge at pH values above the isoelectric point. The hydrophilic tertiary amine head group of CTAB may be electrostatically attracted to the oppositely charged amino acid residues of the enzyme. Also, there might be hydrophobic interactions between the alkyl chains of the surfactant and the hydrophobic residues of the enzyme. These interactions can induce conformational changes that led to the enzyme inactivation.

Another important factor that may induce conformational changes on the enzyme molecules in reverse micelles is the atypical properties of the encapsulated water. Two different populations of water molecules have been identified inside the reverse micelles, namely highly immobile water attached to the polar head-groups of the surfactant molecules and the water molecules that are located in the inner part of the reverse micelle with the same properties as the bulk molecules. In small reverse micelles formed by cationic surfactants, water has been found to attach to the surfactant layer and show different physicochemical properties, compared to bulk water, which may consequently affect the enzyme conformation. 

In this investigation, the influence of oil type on the enzyme activity was also evaluated. The maximum and the minimum laccase activity were seen in microemulsions containing octane (or *iso*-octane) and cyclohexane as oil phase, respectively. These results suggest that the hydrophobicity of the oil could have a strong impact on the enzyme activity. Octanol-water partition coefficient (log P) is used as a measure of molecular hydrophobicity. Among the oils applied, octane and *iso*-octane have the highest log P values (4.78 and 4.37, respectively) and therefore the highest hydrophobicity, whereas cyclohexane shows the lowest log P (3.41) and hence lowest hydrophobicity. It is expected that in the presence of oils with more hydrophobicity in the external phase, the enzyme is forced to immigrate into the water core of the microemulsion droplets.

## Conclusions

As a consequence of many potential advantages, including transparency, thermodynamic stability, large intrinsic interfacial surface, ease of preparation, nanometer-sized aqueous compartments and capability of protecting enzyme from the unfavorable effects of the surrounding, water-in-oil microemulsions have found practical applications as media for enzyme-catalyzed reactions. Our results demonstrated, however, a much lower laccase activity in CTAB-based microemulsions as compared to that in aqueous citrate buffer without any surfactant, thus confirming the deactivation effect of the cationic CTAB on the enzyme. This effect might be related to the conformational change of the enzyme, due to the possible laccase-CTAB interactions that makes the enzyme more inactivated. Mechanism for such deactivation is worth further investigation.
